# Association of thyroid hormone sensitivity indicators with visceral fat area in euthyroid overweight/obese type 2 diabetes patients: sex differences

**DOI:** 10.3389/fendo.2025.1699552

**Published:** 2025-11-20

**Authors:** Xuan Ma, Qingyi Zhou, Chen Ma, Jie Sheng, Xinghe Jiang, Guanqi Gao, Baolan Ji

**Affiliations:** 1 School of Clinical Medicine, Shandong Second Medical University, Weifang, Shandong, China; 2 Department of Endocrinology, Linyi People’s Hospital Affiliated to Shandong Second Medical University, Linyi, Shandong, China; 3 Second Affiliated Hospital, Bengbu Medical University, Bengbu, Anhui, China; 4 School of Chinese Medicine, Beijing University of Chinese Medicine, Beijing, China

**Keywords:** thyroid hormone sensitivity, visceral fat area, visceral fat obesity, type 2 diabetes mellitus, sex differences

## Abstract

**Aims:**

Thyroid hormone (TH) sensitivity plays a key role in glucose and lipid metabolism. However, its relationship with visceral fat distribution in euthyroid overweight/obese patients with type 2 diabetes mellitus (T2DM) remains unclear. This study aimed to examine the associations between TH sensitivity indices and visceral fat area (VFA), with particular attention to sex-specific differences.

**Methods:**

A total of 831 euthyroid overweight/obese T2DM patients (374 males and 457 females) were enrolled. Multiple TH sensitivity indices were calculated. VFA was measured by bioelectrical impedance analysis (BIA), and visceral fat obesity (VFO) was defined as VFA ≥ 100 cm². Associations were assessed using univariate and multivariate linear and logistic regression models, adjusting for age, BMI, and glycemic control parameters.

**Results:**

In males, TFQI_FT3_ was independently associated with VFA (β = 0.088, P < 0.05) and predicted VFO (OR: 2.545; 95% CI: 1.206–5.370, P = 0.014). In females, no significant associations were observed between TH sensitivity indices and either VFA or VFO, indicating a clear sex-specific difference.

**Conclusions:**

TFQI_FT3_ may serve as a potential marker linking thyroid hormone sensitivity with visceral fat accumulation, particularly in male T2DM patients.

## Introduction

Overweight/obesity are major risk factors for type 2 diabetes mellitus (T2DM), a growing global health challenge (1). Among obesity phenotypes, abdominal obesity characterized by increased visceral fat area (VFA) is particularly associated with the development and complications of T2DM, as well as with hypertension, cardiovascular disease, and other metabolic disorders (2). Abdominal (central) fat includes both subcutaneous and visceral adipose tissue, but visceral fat—located around intra-abdominal organs—has greater metabolic activity and is more strongly linked to insulin resistance, chronic low-grade inflammation and cardiometabolic risk (3). VFA specifically quantifies visceral adiposity, providing a more accurate assessment of metabolically active fat than anthropometric measures such as waist circumference (4). Beyond these effects, obesity and insulin resistance may also alter the hypothalamic–pituitary–thyroid axis and deiodinase activity, leading to reduced tissue responsiveness to thyroid hormones despite normal circulating levels (5, 6). Such diminished thyroid hormone sensitivity may further impair lipid mobilization and energy expenditure, thereby exacerbating visceral fat accumulation and metabolic dysfunction in T2DM (7). Motivated by these considerations, we examined sex-specific associations between thyroid hormone sensitivity indicators and VFA in euthyroid overweight/obese adults with T2DM.

Thyroid hormones (THs), including free triiodothyronine (FT3), free thyroxine (FT4), and thyroid-stimulating hormone (TSH), are central regulators of energy and lipid metabolism, influencing basal metabolic rate, lipid profiles, and fat accumulation (8). While overt or subclinical hypothyroidism has been extensively studied in the context of obesity (9), emerging evidence indicates that subtle variations in TH function and sensitivity are closely associated with visceral fat area (VFA) in euthyroid individuals (10, 11).

Some overweight/obese patients with T2DM may exhibit reduced thyroid hormone (TH) sensitivity, or mild TH resistance, which could contribute to metabolic disturbances despite normal thyroid function (12–14). Various indices have been developed to assess central and peripheral TH sensitivity, such as the FT3/FT4 ratio, TSH index (TSHI), thyrotroph T3 and T4 resistance indices (TT3RI, TT4RI), and thyroid feedback quantile-based indices (TFQI_FT3_, TFQI_FT4_). These indices have been linked to diabetes and its complications (12, 15, 16); however, their relationship with VFA in euthyroid overweight/obese T2DM patients remains unclear, and potential sex differences in this association have not been fully explored.

Based on these shared metabolic mechanisms, we hypothesized that thyroid hormone sensitivity is closely related to visceral fat accumulation in T2DM and may exhibit sex-specific differences. Therefore, this study aimed to investigate the associations between TH sensitivity indices and VFA in euthyroid overweight/obese patients with T2DM and to determine potential sex-specific patterns. These findings may help clarify the metabolic role of thyroid hormones in obesity-related diabetes and support more precise risk assessment and management strategies.

## Materials and methods

### Study participants

**Figure 1:**
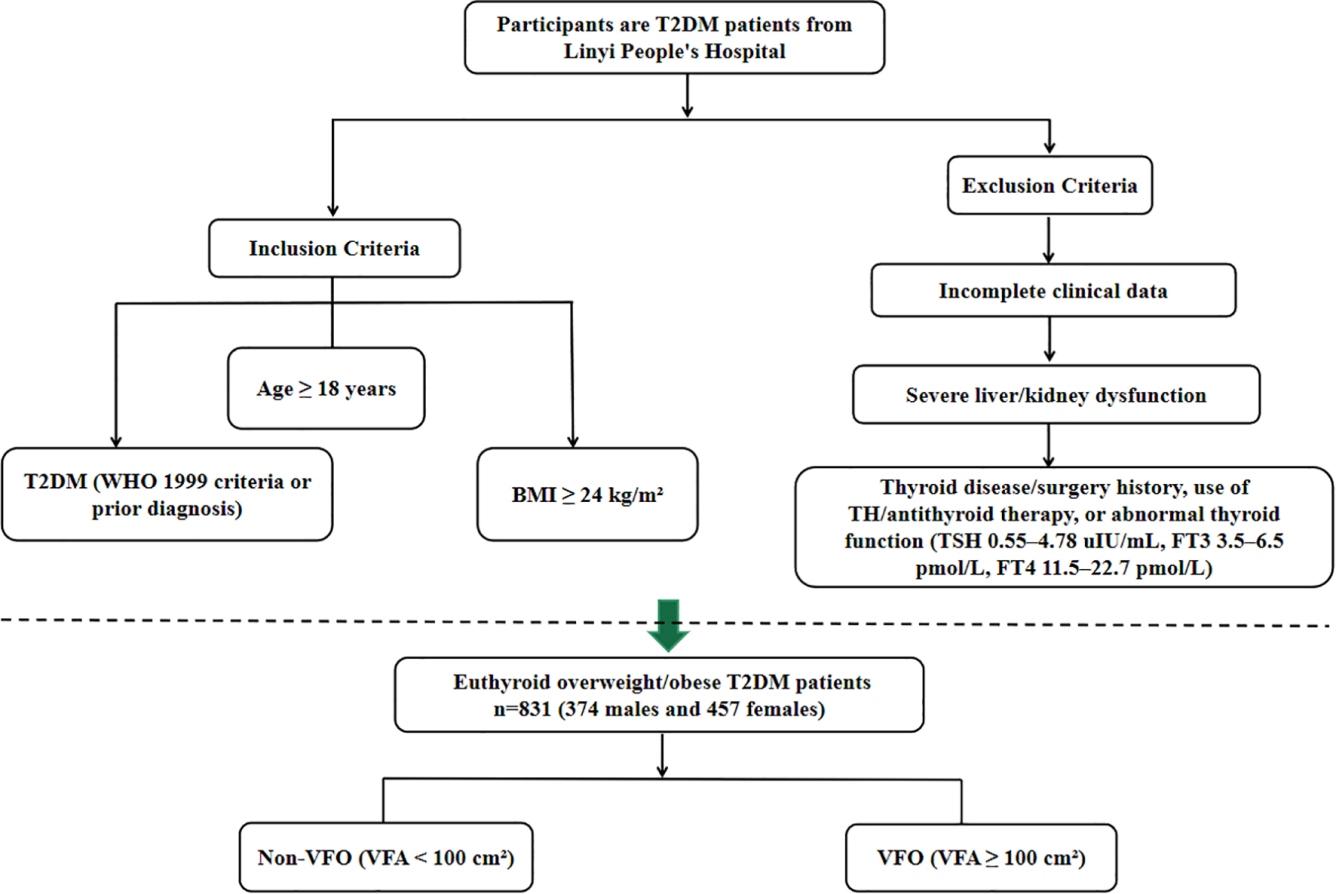
Flowchart showing the selection of euthyroid overweight/obese patients with type 2 diabetes mellitus from Linyi People’s Hospital.

Medical records of patients with T2DM treated at the Department of Endocrinology, Linyi People’s Hospital, between January 2020 and March 2023 were reviewed. Inclusion criteria were: (1) a diagnosis of T2DM according to the 1999 WHO criteria or a prior confirmed diagnosis of T2DM; (2) age ≥ 18 years; and (3) BMI ≥ 24 kg/m². According to the *Guidelines for Prevention and Control of Overweight and Obesity in Chinese Adults*, BMI of 24.0–27.9 kg/m² was defined as overweight and BMI ≥ 28.0 kg/m² as obesity; therefore, participants with BMI ≥ 24.0 kg/m² were categorized as overweight/obese in this study. Patients were excluded if they had: (1) incomplete clinical data; (2) a history of thyroid disease or surgery, use of TH replacement or antithyroid therapy, or abnormal thyroid function (TSH 0.55–4.78 uIU/mL, FT3 3.5–6.5 pmol/L, FT4 11.5–22.7 pmol/L); or (3) severe liver or kidney dysfunction. After screening, 831 euthyroid overweight/obese T2DM patients were included (374 males and 457 females). Participants were categorized according to VFA into a non-visceral fat obesity group (Non-VFO, VFA <100 cm²) and a visceral fat obesity group (VFO, VFA ≥100 cm²) (17). The overall patient selection process is summarized in **Figure 1**.

### Medical data and biochemical measurements

Demographic and clinical data, including age, sex, diabetes duration, and self-reported smoking and alcohol consumption status, were obtained from medical records. Anthropometric measurements, including height, weight, and systolic and diastolic blood pressures (SBP and DBP), were recorded following standardized protocols.

After an overnight fast, venous blood samples were collected in the morning to assess biochemical and metabolic parameters, including fasting blood glucose (FBG); lipid profile, comprising total cholesterol (TC), triglycerides (TG), high-density lipoprotein cholesterol (HDL-c), and low-density lipoprotein cholesterol (LDL-c); liver function markers, including alanine aminotransferase (ALT), aspartate aminotransferase (AST), and γ-glutamyl transferase (GGT); renal function indicators, including serum creatinine (Scr) and uric acid (UA); hemoglobin (Hb); and glycated hemoglobin (HbA1c), measured by high-performance liquid chromatography.

Urinary creatinine and microalbumin (UMA) were measured using an automated analyzer (Beckman Coulter AU5821) via the picric acid and turbidimetric methods, respectively, and the urine albumin-to-creatinine ratio (UACR) was calculated. Thyroid function, including free triiodothyronine (FT3), free thyroxine (FT4), thyroid-stimulating hormone (TSH), and anti-thyroid peroxidase antibody (TPOAb), was measured using chemiluminescence immunoassay (SIEMENS, USA), in line with the manufacturer’s reference interval (0–60 IU/mL), TPOAb positivity was defined as >60 IU/mL.

### Parameter calculations

1. BMI = weight (kg)/height^2^ (m^2^);
2. FT3/FT4 ratio = FT3 (pmol/L)/FT4 (pmol/L);
3. TFQI_FT3_ = cdf FT3 − (1 − cdf TSH) (18);
4. TFQI_FT4_ = cdf FT4 − (1 − cdf TSH) (13);
5. TSHI = ln TSH (mIU/L) + 0.1345 × FT4 (pmol/L) (19);
6. TT3RI = FT3 (pmol/L) × TSH (mIU/L) (20);
7. TT4RI = FT4 (pmol/L) × TSH (mIU/L) (21).

### Visceral fat area

VFA was measured using a bioelectrical impedance analyzer (BIA, Omron DUALSCAN HDS-2000, Kyoto, Japan). Participants rested in the supine position for at least 5 minutes prior to the assessment. During breath-holding, an abdominal measuring unit was applied, and conductive gel was placed on eight electrode pads. An electrode belt was fastened around the waist and connected to clamp electrodes attached to both hands and feet (two per limb). The device quantified the total abdominal cross-sectional area, subcutaneous fat area, and excluded regions such as muscles and internal organs. VFA was then calculated by subtracting the non-fat and subcutaneous fat areas from the total abdominal cross-sectional area.

VFA was analyzed both as a continuous variable and as a categorical variable (VFO vs. Non-VFO) to evaluate the consistency and clinical relevance of associations between TH sensitivity indices and visceral fat accumulation. The continuous analysis allowed assessment of quantitative dose-response relationships, while the categorical analysis facilitated clinical interpretation and risk stratification.

### Sample size and power considerations

As this was a retrospective cross-sectional study of a fixed cohort, no *a priori* sample-size calculation was performed. All eligible participants during the study period were consecutively enrolled (January 2020–March 2023; n=831; 374 men, 457 women). To assess statistical adequacy, we computed achieved power for the primary male linear model (VFA regressed on TFQI_FT3_) using the observed coefficient and standard error under a two-sided α=0.05; achieved power exceeded 0.80. For the sex-stratified logistic regressions of VFO, model stability was verified using the events-per-variable (EPV) criterion: with 267 male and 199 female VFO events and five parameters per model, EPV was 53.4 (men) and 39.8 (women), well above the conventional threshold of 10.

### Statistical analysis

Statistical analyses were performed using SPSS version 25.0 (SPSS Inc., Chicago, IL, USA). Data with a normal distribution are presented as mean ± standard deviation, whereas skewed data are expressed as median (interquartile range). Differences between groups were assessed using the independent-samples to test for normally distributed variables and the Mann–Whitney U test for non-normally distributed variables. Categorical variables were compared using the chi-square test.

Because all TH indices derive from TSH, FT4, and FT3, we analyzed each index separately in parallel, sex-specific multivariable models with identical covariates (indices not co-entered). Collinearity was assessed by tolerance/VIF (VIF >5 moderate, >10 severe) and cross-index relatedness by correlation matrices; models used the Enter method with listwise deletion. Given the metabolic relevance of autoimmunity, TPOAb was prespecified as a male effect modifier: we reported prevalence, examined correlations with VFA/VFO, fitted VFA models stratified by TPOAb (>60 vs ≤60 IU/mL), and tested TFQI_FT3_×TPOAb. The small positive stratum (n=12) was modeled parsimoniously with TFQI_FT3_ forced in.

Pearson’s or Spearman’s correlation coefficients were calculated to evaluate associations between TH sensitivity indices and VFA. Multivariate linear regression was applied to identify independent factors associated with VFA, while stepwise binary logistic regression was used to determine independent predictors of VFO. A two-sided p < 0.05 was considered statistically significant.

### Ethical approval

This study was conducted in accordance with the Declaration of Helsinki and was approved by the Ethics Committee of Linyi People’s Hospital (approval number: 202404-H-018, approval date: April 2024). Written informed consent was obtained from all participants prior to enrollment.

## Results

### Baseline characteristics

A total of 831 euthyroid overweight/obese T2DM patients were included, comprising 374 males (45.0%) and 457 females (55.0%). Among them, 466 (56.1%) were classified as having VFO (VFA ≥ 100 cm²). The mean age was 55.6 ± 12.4 years in males and 59.8 ± 13.6 years in females, median diabetes duration was 8 (3–12) years, mean BMI was 27.2 ± 2.9 kg/m², and mean HbA1c was 9.1 ± 2.0%.

Overall, participants with VFO had higher blood pressure, BMI, SFA, liver enzymes (ALT, AST, GGT), TG, and UACR, and lower HDL-c compared with non-VFO participants. Detailed baseline parameters are summarized in **Table 1**.

**Table 1:** Comparison of baseline clinical characteristics between the VFO and Non-VFO groups stratified by sex.

Variables	Male			Female		
	VFO groups	Non-VFO groups	P	VFO groups	Non-VFO groups	P
Number	267	107		199	258	
Demographics						
Age (years)	55.6 ± 12.4	56.4 ± 10.6	0.520	59.8 ± 13.6	57.9 ± 10.4	0.100
Duration of diabetes (years)	8(3,12)	9(3,12)	0.741	8(2 ,14)	7.5(3 ,12)	0.869
Smoking (%)	121 (45.3%)	36 (33.6%)	1.000	1 (5%)	1 (4%)	0.943
Alcohol consumption (%)	106 (39.7%)	33 (33.8%)	0.274	1 (4%)	0 (0%)	0.765
Biochemical indices						
SBP (mmHg)	133.3 ± 18.0	126.4 ± 15.9	0.001	135.9 ± 20.9	130.0 ± 18.7	0.001
DBP (mmHg)	84.5 ± 11.4	80.9 ± 10.0	0.004	81.3 ± 12.2	78.9 ± 9.9	0.028
Hb (g/L)	154.60 ± 14.66	150.37 ± 13.57	0.010	135.80 ± 13.24	134.93 ± 13.87	0.496
Scr (μ/mol/L)	73.24 ± 20.11	74.40 ± 18.72	0.608	57.70 ± 15.11	54.27 ± 12.80	0.009
UA (μ/molL)	328.06 ± 84.61	312.41 ± 75.58	0.082	288.51 ± 88.33	249.18 ± 74.26	<0.001
AST (U/L)	18.60 (15.50 ,24.60)	17.10 (13.40 ,19.50)	0.001	17.80 (14.50 ,23.70)	16.30 (13.58 ,19.63)	0.002
ALT (U/L)	22.80 (16.80 ,36.10)	17.40 (14.00 ,22.80)	<0.001	18.40 (13.50 ,26.90)	16.00 (12.28 ,22.08)	0.002
GGT (U/L)	30.00 (22.00 ,44.00)	23.00 (17.30 ,32.00)	<0.001	22.00 (16.00 ,32.00)	18.00 (14.00 ,26.00)	<0.001
TG (mmol/L)	1.72 (1.23 ,2.52)	1.33 (0.86 ,1.98)	<0.001	1.63 (1.14 ,2.44)	1.43 (1.10 ,1.97)	0.013
TC (mmol/L)	4.54 ± 1.31	4.33 ± 1.14	0.157	4.91 ± 1.22	4.95 ± 1.17	0.733
HDL-c (mmol/L)	1.00 ± 0.21	1.07 ± 0.24	0.009	1.17 ± 0.27	1.24 ± 0.39	0.021
LDL-c (mmol/L)	2.79 ± 1.00	2.77 ± 0.96	0.829	3.07 ± 1.03	3.16 ± 0.95	0.338
UACR (U/L)	9.70 (4.60 ,48.60)	9.20(5.80 ,40.20)	<0.001	13.60 (7.00 ,33.40)	9.35 (5.78 ,27.02)	0.001
FBG (mmol/L)	8.67 ± 3.06	8.87 ± 3.07	0.584	8.81 ± 3.69	9.66 ± 3.78	0.016
HbA1c (%)	9.19 ± 2.06	8.92 ± 1.84	0.288	9.12 ± 2.06	9.44 ± 1.93	0.092
Anthropometric measures						
BMI (kg/m2)	27.76 ± 2.55	25.86 ± 1.40	<0.001	28.97 ± 3.63	26.28 ± 1.75	<0.001
SFA (cm2)	227.53 ± 51.02	174.49 ± 34.28	<0.001	246.47 ± 70.55	200.38 ± 52.75	<0.001
Thyroid hormone indices						
FT3 (Pmol/L)	5.02 ± 0.55	4.83 ± 0.54	0.002	4.60 ± 0.50	4.52 ± 0.47	0.103
FT4 (Pmol/L)	16.52 ± 2.07	16.34 ± 2.17	0.440	15.68 ± 1.93	15.52 ± 2.12	0.412
FT3/FT4 ratio	0.31 ± 0.05	0.30 ± 0.05	0.172	0.30 ± 0.04	0.30 ± 0.05	0.913
TSH (uIU/mL)	1.68 (1.21 ,2.29)	1.44 (1.01 ,2.37)	0.081	1.87 (1.42 ,2.79)	1.94 (1.33 ,2.83)	0.917
TPOAb (IU/mL)	36.80 (32.50 ,45.10)	37.40 (33.60 ,41.60)	0.946	37.05 (31.88 ,45.03)	38.35 (33.40 ,46.00)	0.163
TPOAb positive (%)	10 (3.7%)	2 (1.9%)	<0.001	18 (7.0%)	14 (7.0%)	<0.001
TSHI	2.74 ± 0.48	2.61 ± 0.57	0.028	2.74 ± 0.53	2.73 ± 0.52	0.748
TT3RI	9.22 ± 4.11	8.22 ± 4.20	0.035	9.59 ± 4.25	9.57 ± 4.48	0.962
TT4RI	30.24 ± 13.58	27.92 ± 14.82	0.146	32.85 ± 15.18	32.54 ± 14.89	0.827
TFQI_FT3_	0.10 ± 0.38	-0.07 ± 0.39	<0.001	0.04 ± 0.36	0.01 ± 0.38	0.429
TFQI_FT4_	-0.02 ± 0.35	-0.10 ± 0.40	0.053	-0.01 ± 0.39	-0.04 ± 0.37	0.392

SBP, systolic blood pressure; DBP, diastolic blood pressure; Hb, hemoglobin; Scr, serum creatinine; UA, uric acid; AST, aspartate aminotransferase; ALT, alanine aminotransferase; GGT, γ-glutamyl transpeptidase; TG, triglycerides; TC, total cholesterol; HDL-C, high-density lipoprotein cholesterol; LDL-C, low-density lipoprotein cholesterol; UACR, urinary albumin-to-creatinine ratio; FBG, fasting blood glucose; HbA1c, glycated hemoglobin; BMI, body mass index; SFA, subcutaneous fat area; FT3, free triiodothyronine; FT4, free thyroxine; TSH, thyroid-stimulating hormone; TPOAb, anti-thyroperoxidase antibody; TSHI, TSH index; TT3RI, thyrotroph T3 resistance index; TT4RI, thyrotroph T4 resistance index; TFQI_FT3_, thyroid feedback quantile-based index calculated by FT3; TFQI_FT4_, thyroid feedback quantile-based index calculated by FT4; VFA, visceral fat area; VFO, visceral fat obesity, defined as VFA ≥ 100 cm²; Data are presented as mean ± standard deviation (SD) for normally distributed variables and as median (interquartile range) for abnormally distributed variables. Independent-samples *t* test and Mann–Whitney *U* test were used for comparisons of normally and abnormally distributed continuous variables between groups, respectively. Categorical variables are presented as frequency (percentage) and compared using the chi-square test. Statistical significance was defined as a two-tailed p-value < 0.05.

Among participants with TPOAb data, 12/236 (5.1%) males and 32/292 (11.0%) females were TPOAb-positive (Fisher’s exact test p=0.017). In males, TPOAb showed no correlation with VFA (r=−0.025, p=0.697) or VFO (r=0.004, p=0.946) (**Supplementary Table S1**).

### Clinical characteristics stratified by sex

When stratified by sex, in males, VFO was associated with higher blood pressure, liver enzymes, TG, UACR, FT3, BMI, SFA, and TH sensitivity indices (FT3, TSHI, TT3RI, TFQI_FT3_), and lower HDL-c. In females, VFO was related to higher blood pressure, renal and liver function markers, TG, UACR, FBG, BMI, and SFA (all P < 0.05); Detailed results are summarized in **Table 1**, and corresponding statistical comparisons (t/Z/χ² and P values) between VFO and non-VFO groups are provided in **Supplementary Table S1**.

### VFA, VFO and correlation analysis

**Figure 2:**
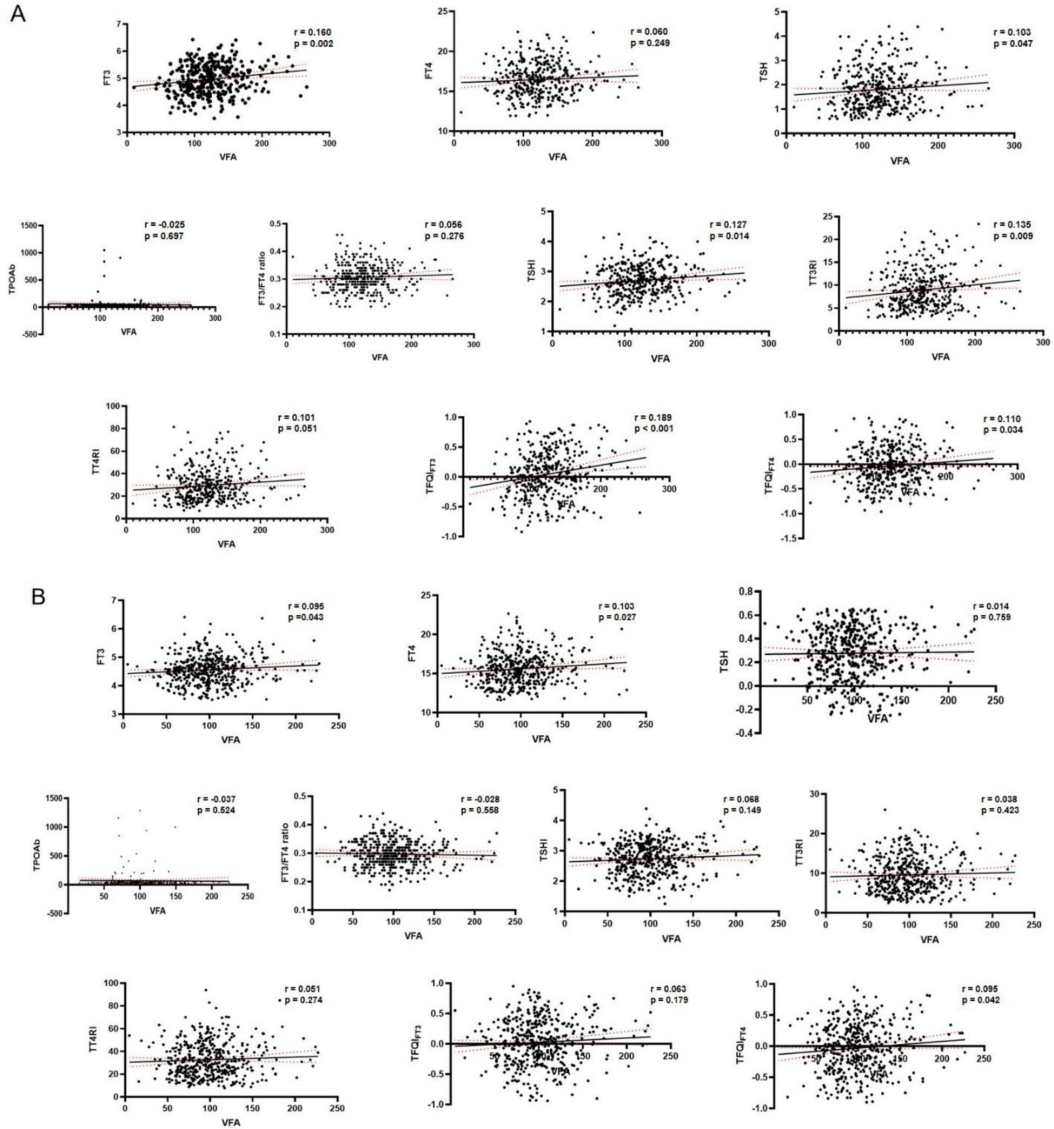
Correlations between visceral fat area (VFA) and thyroid hormone–related indices by sex.

Pearson correlation analysis indicated that in males, VFA was positively correlated with blood pressure, liver enzymes, TG, FT3, BMI, SFA, and TH sensitivity indices (TSHI, TT3RI, TFQI_FT3_, TFQI_FT4_), and negatively with HDL-c (all P < 0.05). In females, VFA correlated positively with blood pressure, UA, UACR, liver enzymes, TG, FBG, HbA1c, FT3, FT4, BMI, SFA, and TFQI_FT4_, but negatively with HDL-c (all P < 0.05). Corresponding scatter plots of the correlations between thyroid hormone indices and VFA are shown in **Figure 2**: (A) male participants and (B) female participants.

Spearman correlation analysis showed that in males, VFO was positively associated with blood pressure, Hb, liver enzymes, TG, UACR, FT3, BMI, SFA, and TH sensitivity indices (TSHI, TT3RI, TT4RI, TFQI_FT3_), and negatively with HDL-c (all P < 0.05). In females, VFO was positively correlated with age, blood pressure, renal indices, liver enzymes, TG, UACR, FBG, BMI, and SFA, while negatively with HDL-c (all P < 0.05) (**Table 2**).

**Table 2:** Univariate analysis of variables associated with VFA and VFO.

Variables	Male				Female			
	VFA		VFO		VFA		VFO	
	Correlation Coefficient	P	Correlation Coefficient	P	Correlation Coefficient	P	Correlation Coefficient	P
Demographics								
Age	0.008	0.875	-0.015	0.777	0.050	0.282	0.132	0.005
Duration of diabetes	0.022	0.676	-0.017	0.741	-0.041	0.379	0.008	0.869
Smoking			0.107	0.039			0.009	0.854
Alcohol consumption			0.083	0.110			-0.041	0.380
Biochemical indices								
SBP	0.221	<0.001	0.185	<0.001	0.146	0.002	0.141	0.003
DBP	0.227	<0.001	0.148	0.004	0.161	0.001	0.099	0.034
Hb	0.093	0.072	0.155	0.003	0.042	0.367	0.031	0.505
Scr	0.081	0.118	-0.030	0.564	0.053	0.262	0.123	0.009
UA	0.194	<0.001	0.083	0.110	0.222	<0.001	0.238	<0.001
AST	0.140	0.007	0.180	<0.001	0.198	<0.001	0.145	0.002
ALT	0.192	<0.001	0.263	<0.001	0.245	<0.001	0.148	0.002
GGT	0.249	<0.001	0.249	<0.001	0.279	<0.001	0.183	<0.001
TG	0.272	<0.001	0.219	<0.001	0.213	<0.001	0.117	0.013
TC	0.084	0.106	0.064	0.216	0.024	0.609	-0.003	0.944
HDL-c	-0.133	0.010	-0.137	0.008	-0.186	<0.001	-0.113	0.015
LDL-c	0.017	0.742	0.013	0.795	-0.009	0.856	-0.029	0.540
UACR	0.058	0.260	-0.022	0.674	0.148	0.001	0.120	0.010
FBG	0.086	0.098	0.036	0.491	0.145	0.002	0.129	0.006
HbA1c	-0.029	0.579	-0.046	0.379	0.111	0.017	0.082	0.080
Anthropometric measures								
BMI	0.636	<0.001	0.380	<0.001	0.602	<0.001	0.429	<0.001
SFA	0.640	<0.001	0.482	<0.001	0.480	<0.001	0.358	<0.001
Thyroid hormone indices								
FT3	0.160	0.002	0.165	0.001	0.095	0.043	0.083	0.076
FT4	0.060	0.249	0.047	0.362	0.103	0.027	0.057	0.227
TSH	0.103	0.047	0.090	0.081	0.014	0.759	-0.005	0.917
TPOAb	-0.025	0.697	0.004	0.946	-0.037	0.524	-0.082	0.163
TPOAb positive	0.032	0.627	0.056	0.389	-0.020	0.737	0.004	0.942
FT3/FT4 ratio	0.056	0.276	0.100	0.054	-0.028	0.558	0.019	0.682
TSHI	0.127	0.014	0.120	0.020	0.068	0.149	0.020	0.669
TT3RI	0.135	0.009	0.127	0.014	0.038	0.423	0.012	0.800
TT4RI	0.101	0.051	0.102	0.048	0.051	0.274	0.007	0.879
TFQI_FT3_	0.189	<0.001	0.207	<0.001	0.063	0.179	0.039	0.402
TFQI_FT4_	0.110	0.034	0.101	0.051	0.095	0.042	0.037	0.428

SBP, systolic blood pressure; DBP, diastolic blood pressure; Hb, hemoglobin; Scr, serum creatinine; UA, uric acid; AST, aspartate aminotransferase; ALT, alanine aminotransferase; GGT, γ-glutamyl transpeptidase; TG, triglycerides; TC, total cholesterol; HDL-C, high-density lipoprotein cholesterol; LDL-C, low-density lipoprotein cholesterol; UACR, urinary albumin-to-creatinine ratio; FBG, fasting blood glucose; HbA1c, glycated hemoglobin; BMI, body mass index; SFA, subcutaneous fat area; FT3, free triiodothyronine; FT4, free thyroxine; TSH, thyroid-stimulating hormone; TPOAb, anti-thyroperoxidase antibody; TSHI, TSH index; TT3RI, thyrotroph T3 resistance index; TT4RI, thyrotroph T4 resistance index; TFQIFT3, thyroid feedback quantile-based index calculated by FT3; TFQIFT4, thyroid feedback quantile-based index calculated by FT4; VFA, visceral fat area; VFO, visceral fat obesity, defined as VFA ≥ 100 cm²; Correlation coefficients between VFA/VFO and different variables were determined using Pearson correlation analysis and Spearman correlation analysis.

In index-specific diagnostics, multicollinearity appeared acceptable (all tolerance >0.10; all VIF <5); in males, TFQI_FT3_ VIF = 1.067 (tolerance=0.937). As expected from their shared TSH/FT4/FT3 basis, the indices were moderately to strongly correlated (males: Pearson |r| 0.160–0.951; females: 0.163–0.934; see **Supplementary Materials**).

### Multiple regression analysis of VFA and VFO

Variables significantly correlated with VFA or VFO in correlation analyses were included in stepwise multivariate linear and logistic regression models. In males, stepwise linear regression showed that TFQI_FT3_ (β = 0.088, SE = 3.568, t = 2.731, P = 0.018), SFA, BMI, TG, and SBP were independently associated with VFA. In females, BMI, GGT, HDL-c, and SBP were independent predictors of VFA (all exact P-values reported in **Table 3**).

**Table 3:** Linear regression analysis identifying independent factors associated with VFA.

Variables	Unstandardized coefficient		Standardized coefficient	t	P
	B	SE	β		
Male					
TFQI_FT3_	8.461	3.568	0.088	2.731	0.018
SFA	0.264	0.039	0.368	6.848	<0.001
BMI	4.822	0.884	0.312	5.714	<0.001
TG	18.075	5.319	0.128	3.398	0.001
SBP	0.227	0.078	0.130	3.537	0.003
Female					
BMI	6.072	0.418	0.550	14.518	<0.001
GGT	18.002	5.569	0.123	3.233	0.001
SBP	0.183	0.062	0.109	2.957	<0.001
HDL-c	-9.838	3.652	-0.101	-2.694	0.007

TFQIFT3, thyroid feedback quantile-based index calculated by FT3; SFA, subcutaneous fat area; BMI, body mass index; TG, triglycerides; SBP, systolic blood pressure; GGT, γ-glutamyl transpeptidase; HDL-C, high-density lipoprotein cholesterol; VFA, visceral fat area; B, unstandardized regression.

In TPOAb-negative males (n=224), TFQI_FT3_ remained associated with VFA (β=9.78; 95% CI 0.50–19.06; p=0.039). In TPOAb-positive males (n=12), a parsimonious Enter model (TFQI_FT3_ forced in) yielded β=−52.92; 95% CI −130.36 - 24.52; p=0.159 (**Supplementary Table S2**). In the full male sample, the TFQI_FT3_×TPOAb interaction was not significant (β=0.084; 95% CI −0.085 - 0.252; p=0.329), suggesting no evidence of effect modification by TPOAb status (**Supplementary Table S3**).

Logistic regression revealed that in males, TFQI_FT3_ (OR = 2.545, 95% CI: 1.206 - 5.370, P = 0.014), TG, ALT, SFA, and SBP were independent predictors of VFO. In females, BMI, GGT, UA, age, and SBP were independently associated with VFO (all P-values and 95% CIs in **Table 4**). Although several associations did not reach statistical significance in females, providing these results allows a complete sex-stratified comparison.

**Table 4:** Logistic regression analysis identifying independent factors associated with VFO.

Variables	B	SE	Wals	P	OR	95.0 % CI for OR
Male						
TFQI_FT3_	0.934	0.381	6.008	0.014	2.545	1.206 - 5.370
TG	0.278	0.116	5.756	0.016	1.321	1.052 - 1.658
ALT	0.047	0.014	11.329	0.001	1.048	1.020 - 1.077
SFA	0.032	0.004	52.910	<0.001	1.032	1.023 - 1.041
SBP	0.023	0.008	7.753	0.005	1.024	1.007 - 1.040
Female						
BMI	0.433	0.054	63.553	<0.001	1.542	1.386 - 1.715
Age	0.045	0.011	17.558	<0.001	1.046	1.024 - 1.068
GGT	0.017	0.007	6.212	0.013	1.017	1.024 - 1.068
SBP	0.013	0.006	4.520	0.033	1.013	1.001 - 1.024
UA	0.005	0.001	11.839	0.001	1.005	1.002 - 1.008

TFQIFT3, thyroid feedback quantile-based index calculated by FT3; SFA, subcutaneous fat area; BMI, body mass index; TG, triglycerides; SBP, systolic blood pressure; GGT, γ-glutamyl transpeptidase; HDL-C, high-density lipoprotein cholesterol; VFA, visceral fat area; B, unstandardized regression coefficient; SE, standard error; β, standardized regression coefficient. The standardized coefficients (β) were determined using multivariate stepwise linear regression analysis.

## Discussion

In this study of euthyroid overweight/obese patients with T2DM, we observed a striking sex-specific association between TH sensitivity and visceral fat accumulation. Specifically, TFQI_FT3_ was independently associated with VFA in male patients, highlighting a potential sex-dependent role of TH sensitivity in visceral adiposity. Previous research investigating this relationship remains limited, particularly in euthyroid T2DM populations, underscoring the novelty and clinical relevance of our findings.

THs play a crucial role in regulating lipid and glucose metabolism and maintaining overall energy homeostasis (22). Even within the euthyroid range, subtle variations in TH levels may exert significant metabolic effects (23, 24). Rather than focusing solely on absolute hormone concentrations, assessing TH sensitivity provides a novel perspective for understanding their role in metabolic regulation. Recent studies have demonstrated that higher TH sensitivity indices are linked to abdominal obesity, hypertriglyceridemia, and hypertension in euthyroid Chinese populations (25), which is consistent with our findings. Moreover, emerging evidence further shows that higher TT3RI and TFQI_FT3_ levels have been linked to central obesity and lower muscle mass specifically in overweight/obese male patients with T2DM, highlighting sex-specific effects of TH sensitivity on body composition (26).

In our study, several indices—including TSHI, TT3RI, TT4RI, TFQI_FT3_, and TFQI_FT4_—were significantly correlated with VFA. After adjusting for confounders, only TFQI_FT3_, an indicator of central TH sensitivity, remained independently associated. TFQI_FT3_ integrates hormone levels with central feedback regulation, capturing aspects of TH responsiveness most relevant to visceral adiposity in overweight/obese patients with T2DM. Multicollinearity analysis confirmed its independent effect. Elevated TFQI_FT3_ reflects high-normal TSH and FT3, potentially compensating for reduced receptor expression in adipose tissue (27). Impaired central sensitivity may affect adipokine secretion and insulin signaling, promoting lipid accumulation (28). These effects are likely more pronounced in men, who have higher visceral fat, lower adiponectin, and greater insulin resistance (29), making them more susceptible to the metabolic consequences of altered TH sensitivity.

However, the relationship between TH sensitivity and abdominal fat distribution remains controversial. For example, one study in euthyroid patients with T2DM reported that impaired central THs was associated with lower VFA (10), which contrasts with our findings. These inconsistencies may stem from differences in study design, population characteristics, and the indices used to evaluate THs. Notably, TFQI_FT3_ reflects central feedback regulation more comprehensively than traditional markers such as FT3/FT4 ratio or TT3RI, which may contribute to the stronger associations observed in our cohort. Furthermore, our study population—euthyroid overweight/obese adults with T2DM—is characterized by pronounced visceral adiposity and insulin resistance, potentially amplifying the metabolic impact of altered THs. Nevertheless, the underlying mechanisms remain incompletely understood, and prospective longitudinal studies are needed to determine whether impaired THs contributes causally to visceral fat accumulation.

Interestingly, the association between TFQI_FT3_ and visceral fat was observed only in males. Consistent with previous studies, higher FT3 levels have been linked to increased visceral adiposity in overweight/obese men (30). A sex-specific effect is biologically plausible: for a given BMI, men carry a higher visceral (rather than subcutaneous) fat fraction, whereas women tend to have more subcutaneous fat, higher adiponectin, and estrogen-related benefits on glucose metabolism, fat distribution, and inflammation (1, 31) Beyond these differences, the androgen axis may further contribute: obesity-associated hypogonadism is tied to visceral adiposity and metabolic syndrome, with low testosterone promoting visceral fat gain and visceral fat feeding back to suppress the HPT axis via inflammation, leptin resistance, and aromatization—a bidirectional loop (32–34). In such a milieu, TFQI_FT3_, a rank-based index sensitive to mismatch between peripheral FT3 signaling and pituitary feedback, may be more readily detectable in men. We could not assess testosterone/SHBG directly and acknowledge this as a limitation and a priority for future work.

At the biochemical level, THs regulate adipose tissue metabolism by promoting lipolysis, mitochondrial biogenesis, and thermogenesis through AMP-activated protein kinase (AMPK) activation and upregulation of uncoupling proteins (28). They also influence deiodinase activity that converts T4 to the metabolically active T3 form in peripheral tissues (35). Reduced TH sensitivity may therefore lower energy expenditure and lipid oxidation, facilitating visceral fat accumulation and insulin resistance (36). Lifestyle factors such as physical activity and diet could further modulate these relationships, although they could not be directly evaluated in the present study.

Because thyroid autoimmunity could confound metabolic phenotypes, we examined TPOAb. Despite a higher prevalence in females, TPOAb was not correlated with VFA/VFO in males; TFQI_FT3_ remained associated with VFA in TPOAb-negative men; and the TFQI_FT3_×TPOAb term was non-significant (**Supplementary Tables S1–S3**). These findings suggest—but do not prove—that the male TFQI_FT3_–VFA link is unlikely to be driven by overt autoimmunity; estimates were imprecise in TPOAb-positive men (n=12).

This study has several limitations. First, the cross-sectional design precludes causal inference; prospective studies are needed. Second, participants were recruited from a single tertiary hospital in China and detailed geographic/medication data were limited, which may affect generalizability. Third, VFA was assessed by BIA rather than CT/MRI; while pragmatic in diabetes care, BIA can be influenced by hydration and body composition. Fourth, lifestyle/behavioral factors (e.g., physical activity, sedentary time, diet, smoking/alcohol) were not fully quantified, leaving potential residual confounding. Finally, unequal sex-stratified subgroup sizes may have reduced statistical power for some analyses.

Despite these limitations, this study also has notable strengths. It included a relatively large, well-characterized cohort of patients with T2DM and performed sex-stratified analyses, allowing for the identification of sex-specific associations. Moreover, the use of multiple indices of TH sensitivity provided a more comprehensive evaluation of TH action beyond conventional hormone levels, offering novel insights into their relationship with visceral adiposity.

## Conclusion

In conclusion, this study demonstrates a significant association between TH sensitivity, particularly TFQI_FT3_, and VFA in euthyroid overweight/obese patients with T2DM, with a clear sex-specific difference observed in males. These findings suggest that TFQI_FT3_ may serve as a potential marker for risk stratification and monitoring of visceral adiposity in male patients, facilitating early identification of individuals at higher metabolic risk. Future longitudinal and mechanistic studies are warranted to further elucidate how THs regulate fat distribution and to inform more precise, sex-specific management strategies in this population.

## Data Availability

The raw data supporting the conclusions of this article will be made available by the authors, without undue reservation.
