# Supplementary material for: Association of thyroid hormone sensitivity indicators with visceral fat area in euthyroid overweight/obese type 2 diabetes patients: sex differences
Source: Front Endocrinol (Lausanne). 2025 Nov 20;16:1699552. doi: 10.3389/fendo.2025.1699552 (PMC12675171; doi:10.3389/fendo.2025.1699552)
Supplement: Supplementary file 1 [file Table1.docx]

### Table S1. TPOAb summary and correlations (definition, prevalence, and male correlations)

| Item | Result |
| --- | --- |
| Assay & cut-off | Chemiluminescence immunoassay (Siemens); TPOAb positive >60 IU/mL (reference 0–60 IU/mL). |
| Prevalence (overall) | 44/528 (8.3%) TPOAb-positive |
| Prevalence (male) | 12/236 (5.1%) TPOAb-positive |
| Prevalence (female) | 32/292 (11.0%) TPOAb-positive |
| Sex difference | Fisher’s exact **test** p = 0.017 |
| Correlation in males: TPOAb vs VFA | r = −0.025, p = 0.697, n = 236 |
| Correlation in males: TPOAb vs VFO | r = 0.004, p = 0.946, n = 236 |

VFA, visceral fat area; VFO, visceral fat obesity. Correlations were assessed using Pearson’s method (two-tailed); for non-normally distributed or ordinal variables, Spearman’s rank correlation (two-tailed) was used. Pairwise deletion for missing data. TPOAb positivity was defined as >60 IU/mL.
