# Supplementary material for: Association of thyroid hormone sensitivity indicators with visceral fat area in euthyroid overweight/obese type 2 diabetes patients: sex differences
Source: Front Endocrinol (Lausanne). 2025 Nov 20;16:1699552. doi: 10.3389/fendo.2025.1699552 (PMC12675171; doi:10.3389/fendo.2025.1699552)
Supplement: Supplementary file 2 [file Table2.docx]

### **Table S2**. Male linear models for VFA stratified by TPOAb (>60 vs ≤60 IU/mL)

| Stratum | Covariates in model | β (TFQIFT3) | 95% CI | p-value | n |
| --- | --- | --- | --- | --- | --- |
| TPOAb− (≤60 IU/mL) | SFA, BMI, SBP (Enter) | 9.78 | 0.50 - 19.06 | 0.039 | 224 |
| TPOAb+ (>60 IU/mL) | Parsimonious Enter (TFQIFT3 forced in) | −52.92 | −130.36 - 24.52 | 0.159 | 12 |

### The positive stratum is very small (n=12); a parsimonious model with TFQIFT3 forced in was used to avoid overfitting. Dependent variable: VFA. Unstandardized coefficients (β); 95% CI as shown; listwise deletion for missing data.
