# Supplementary material for: Association of thyroid hormone sensitivity indicators with visceral fat area in euthyroid overweight/obese type 2 diabetes patients: sex differences
Source: Front Endocrinol (Lausanne). 2025 Nov 20;16:1699552. doi: 10.3389/fendo.2025.1699552 (PMC12675171; doi:10.3389/fendo.2025.1699552)
Supplement: Supplementary file 3 [file Table3.docx]

### **Table S3**. Male interaction model (formal test of effect modification by TPOAb)

| Predictor (full male sample, n=236) | β | 95% CI | p-value | Notes |
| --- | --- | --- | --- | --- |
| TFQIFT3 × TPOAb | 0.084 | −0.085 - 0.252 | 0.329 | No significant interaction |
| SFA | 0.315 | 0.210 - 0.420 | <0.001 | Adjuster |
| BMI | 4.055 | 1.905 - 6.205 | <0.001 | Adjuster |

Linear regression with VFA as the dependent variable; Enter method; listwise deletion. TPOAb coded as 1 for >60 IU/mL and 0 for ≤60 IU/mL. The model included the main effects of TFQIFT3 and TPOAb; only the interaction term is displayed here. Thyroid indices were not co-entered in the same model.
