# Supplementary material for: Association of thyroid hormone sensitivity indicators with visceral fat area in euthyroid overweight/obese type 2 diabetes patients: sex differences
Source: Front Endocrinol (Lausanne). 2025 Nov 20;16:1699552. doi: 10.3389/fendo.2025.1699552 (PMC12675171; doi:10.3389/fendo.2025.1699552)
Supplement: Supplementary file 4 [file Table4.docx]

| **Table S4**. Multicollinearity diagnostics (Tolerance and VIF) for VFA models | | | | | | |
| --- | --- | --- | --- | --- | --- | --- |
| Male | | |  | Female | | |
| Variables | Tolerance | VIF |  | Variables | Tolerance | VIF |
| SBP  DBP  Hb  AST  ALT  GGT  TG  HDL-c  UACR  BMI  SFA  FT3  TSHI  TT3RI  TFQIFT3 | 0.520  0.506  0.801  0.285  0.297  0.723  0.781  0.789  0.899  0.430  0.445  0.894  0.903  0.922  0.937 | 1.922  1.978  1.249  3.510  3.363  1.382  1.281  1.268  1.112  2.327  2.249  1.118  1.107  1.085  1.067 |  | SBP  DBP  Scr  UA  AST  ALT  GGT  TG  HDL-c  UACR  FBG  BMI  SFA | 0.661  0.621  0.760  0.700  0.317  0.288  0.736  0.800  0.839  0.883  0.904  0.433  0.471 | 1.513  1.610  1.316  1.429  3.154  3.472  1.358  1.251  1.192  1.133  1.107  2.307  2.123 |

SBP, systolic blood pressure; DBP, diastolic blood pressure; Hb, hemoglobin; AST, aspartate aminotransferase; ALT, alanine aminotransferase; GGT, γ-glutamyl transferase; TG, triglycerides; HDL-c, high-density lipoprotein cholesterol; UACR, urine albumin-to-creatinine ratio; Scr, serum creatinine; UA, uric acid; FBG, fasting blood glucose; BMI, body mass index; SFA, subcutaneous fat area; VFA, visceral fat area. VIF, variance inflation factor, All tolerance >0.10; all VIF <5 (VIF>5 moderate, >10 severe).
