# Supplementary material for: Association of thyroid hormone sensitivity indicators with visceral fat area in euthyroid overweight/obese type 2 diabetes patients: sex differences
Source: Front Endocrinol (Lausanne). 2025 Nov 20;16:1699552. doi: 10.3389/fendo.2025.1699552 (PMC12675171; doi:10.3389/fendo.2025.1699552)
Supplement: Supplementary file 5 [file Table5.docx]

### **Table S5**. Pearson correlation matrix among thyroid sensitivity indices (sex-specific)

#### A. Males (N = 374)

|  | FT3/FT4 ratio | TSHI | TT3RI | TT4RI | TFQIFT3 | TFQIFT4 |
| --- | --- | --- | --- | --- | --- | --- |
| FT3/FT4 ratio | 1.00 |  |  |  |  |  |
| TSHI | −0.344 | 1.00 |  |  |  |  |
| TT3RI | 0.178 | 0.808 | 1.00 |  |  |  |
| TT4RI | −0.160 | 0.926 | 0.928 | 1.00 |  |  |
| TFQIFT3 | 0.473 | 0.602 | 0.788 | 0.628 | 1.00 |  |
| TFQIFT4 | −0.479 | 0.951 | 0.654 | 0.814 | 0.515 | 1.00 |

#### B. Females (N = 457)

|  | FT3/FT4 ratio | TSHI | TT3RI | TT4RI | TFQIFT3 | TFQIFT4 |
| --- | --- | --- | --- | --- | --- | --- |
| FT3/FT4 ratio | 1.00 |  |  |  |  |  |
| TSHI | −0.342 | 1.00 |  |  |  |  |
| TT3RI | 0.166 | 0.816 | 1.00 |  |  |  |
| TT4RI | −0.163 | 0.930 | 0.932 | 1.00 |  |  |
| TFQIFT3 | 0.432 | 0.604 | 0.787 | 0.639 | 1.00 |  |
| TFQIFT4 | −0.509 | 0.934 | 0.644 | 0.810 | 0.510 | 1.00 |

TSHI, TSH index; TT3RI, thyrotroph T3 resistance index; TT4RI, thyrotroph T4 resistance index; TFQIFT3, thyroid feedback quantile-based index calculated by FT3; TFQIFT4, thyroid feedback quantile-based index calculated by FT4; Pearson correlations (two-tailed); missing data handled by pairwise deletion. All coefficients are p < 0.01. Indices derive from TSH, FT4, and FT3 and were not co-entered in the same regression model to avoid collinearity.
